# Optical Properties of Composites Based on Poly(o-phenylenediamine), Poly(vinylenefluoride) and Double-Wall Carbon Nanotubes

**DOI:** 10.3390/ijms22158260

**Published:** 2021-07-31

**Authors:** Mihaela Baibarac, Monica Daescu, Elena Matei, Daniela Nastac, Oana Cramariuc

**Affiliations:** 1 Laboratory of Optical Processes in Nanostructured Materials, National Institute of Materials Physics, Atomistilor Street 405A, POB MG 7, 077125 Bucharest, Romania; monica.daescu@infim.ro; 2 Multifunctional Materials and Structures Laboratory, National Institute of Materials Physics, Atomistilor Street 405A, 077125 Magurele, Romania; elena.matei@infim.ro; 3 IT Centre for Science and Technology, 25 no. Av. Radu Beller Str., 011702 Bucharest, Romania; daniela.c.nastac@gmail.com (D.N.); oanacramariuc@yahoo.com (O.C.)

**Keywords:** poly(o-phenylenediamine), poly(vinylidene fluoride), carbon nanotubes, photoluminescence, Raman scattering, IR spectroscopy

## Abstract

In this work, synthesis and optical properties of a new composite based on poly(o-phenylenediamine) (POPD) fiber like structures, poly(vinylidene fluoride) (PVDF) spheres and double-walled carbon nanotubes (DWNTs) are reported. As increasing the PVDF weight in the mixture of the chemical polymerization reaction of o-phenylenediamine, the presence of the PVDF spheres onto the POPD fibers surface is highlighted by scanning electron microscopy (SEM). The down-shift of the Raman line from 1421 cm^−1^ to 1415 cm^−1^ proves the covalent functionalization of DWNTs with the POPD-PVDF blends. The changes in the absorbance of the IR bands peaked around 840, 881, 1240 and 1402 cm^−1^ indicate hindrance steric effects induced of DWNTs to the POPD fiber like structures and the PVDF spheres, as a consequence of the functionalization process of carbon nanotubes with macromolecular compounds. The presence of the PVDF spheres onto the POPD fiber like structures surface induces a POPD photoluminescence (PL) quenching process. An additional PL quenching process of the POPD-PVDF blends is reported to be induced in the presence of DWNTs. The studies of anisotropic PL highlight a change of the angle of the binding of the PVDF spheres onto the POPD fiber like structures surface from 50.2° to 38° when the carbon nanotubes concentration increases in the POPD-PVDF/DWNTs composites mass up to 2 wt.%.

## 1. Introduction

In the last ten years, poly(o-phenylenediamine) (POPD) was intensively studied, with several applications being reported in the following area: (i) fuel cells [1], (ii) sensors [2,3], (iii) solar cells [4], lithium-ion batteries [5] and so on. Depending on the oxidizing agents used in chemical polymerization, i.e., K_2_Cr_2_O_7_ and HCl, CuCl_2_, (NH_4_)_2_S_2_O_8_, H_2_O_2_ or FeCl_3,_ the POPD morphological structures of the type of ellipsoidal particles [6], micro-belts [7], quantum dots [8], fiber like microstructures with square prism shapes [9] and microfibers [10], respectively, were reported. A wide range of POPD-based composites have been synthesized in the presence of various oxides (e.g., TiO_2_ [11], ZnO [12]) or carbon nanoparticles (e.g., carbon nanohorns [13], reduced graphene oxide [14], carbon nanohollow [15], carbon nanotubes [16,17]). The composites based on POPD and various carbon nanoparticles were intensely studied for a wide range of applications. In order to support this sentence, we note that in the case of: (i) the POPD/graphene composites, the applications in the field of supercapacitors [18,19] and sensors [20] were reported, while in the case of (ii) the POPD/carbon nanotube composites, the main applications were as (a) sensorial platforms for detection of glucose [21], meldonium [22], paraquat [23], pentose [24] and ampicillin [25], (b) corrosion protection layers [26] and (c) as active materials in rechargeable lithium batteries [27] and supercapacitors [28]. Special attention was given to blends consisting of conducting and insulating polymers such as POPD/poly(vinyl alcohol), applications in the sensors field being reported in this particular case [29].

All these applications were possible as a consequence of good knowledge of the optical and structural properties of these materials. In this context, the main experimental techniques used to evidence the optical and structural properties were: scanning electron microscopy (SEM), Raman scattering, Fourier-transform infrared (FTIR) spectroscopy, photoluminescence, X-ray diffraction, UV-VIS spectroscopy and transmission electron microscopy [13,17,21,24].

In contrast with this progress, in this work, the synthesis and the optical properties of the composites based on POPD fiber like structures, poly(vinylidene fluoride) (PVDF) spheres and double-walled carbon nanotubes (DWNTs) are studied. Interest in the addition of insulating polymers such as poly(vinyl alcohol), poly(ethylene oxide) and PVDF to the POPD matrix has been reported for both electro-spinning processes and sensors used in the field of health and the environment [17,29]. In this work, the presence of PVDF spheres on the POPD fiber like structures surface as well as of DWNTs will be shown by scanning electron microscopy (SEM). The optical methods often used to elucidate potential interactions between macromolecular compounds and carbon nanotubes are Raman scattering and IR spectroscopy. In order to highlight the chemical interactions between the POPD-PVDF blends and DWNTs, the vibrational properties of these materials will be presented by Raman scattering and FTIR spectroscopy. Knowledge of the photoluminescent properties of composites based on polymers and carbon nanotubes has been a topic intensively studied in the last ten years for potential applications in the field of energy conversion [30]. Considering the importance of these properties, in this paper, the influence of the PVDF spheres as well as DWNTs on the photoluminescence (PL) of the POPD fiber like structures, will also be reported. Using anisotropic PL, an estimation of the angle of the binding of (i) the PVDF spheres onto the POPD fiber like structures surface and (ii) DWNTs onto POPD-PVDF blends surface will be carried out, too.

## 2. Results

**Figure 1:**
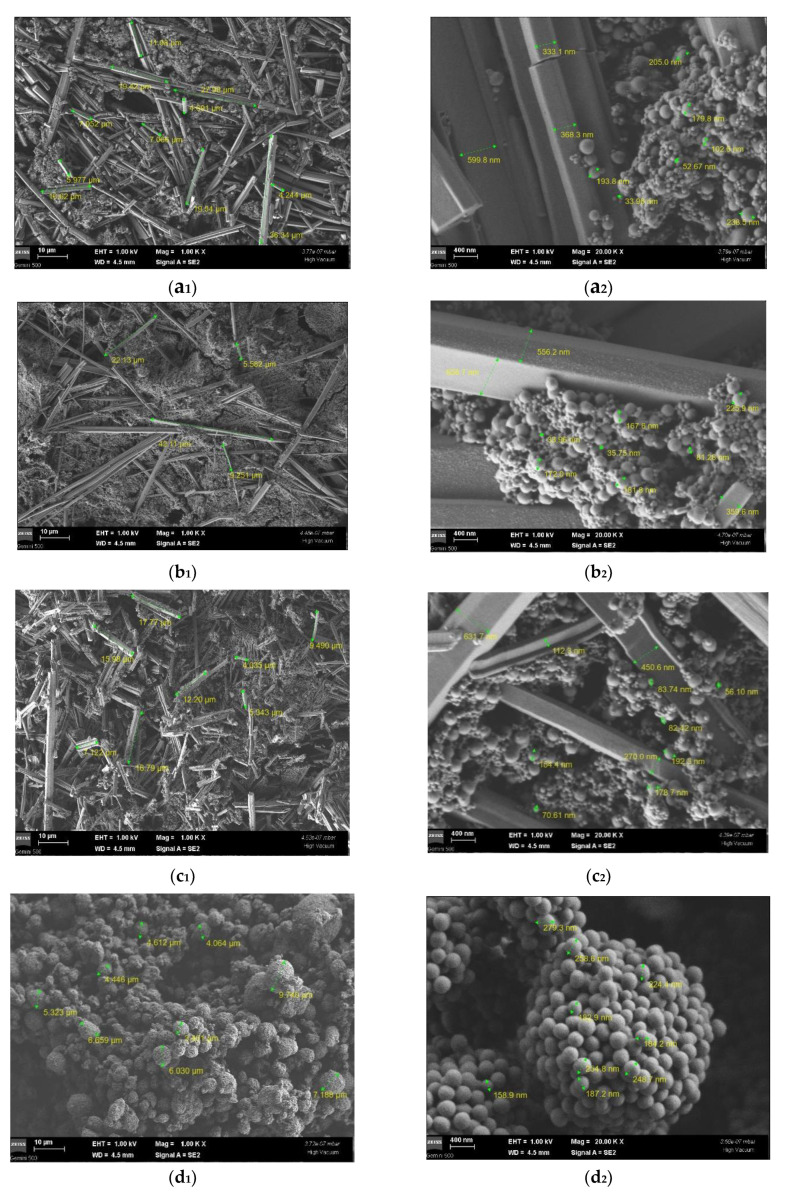
SEM images of the blends based on the POPD fiber like structures and the PVDF spheres corresponding to the A (**a**), B (**b**) and C (**c**) samples. (**d**) shows the PVDF spheres. (**a_1_**–**d_1_**) show SEM images with magnification of × 1.00K, while in the case of (**a_2_**–**d_2_**), SEM images with magnification × 20.00K are shown.

Figure 1 shows the SEM images of the A, B and C samples as well as PVDF. As can be seen in Figure 1d_1_,d_2_, PVDF has the shape of spheres with a diameter between 160 and 280 nm, the spheres being arranged in aggregates with the size around 3–10 µm. Figure 1a_1_–c_1_,a_2_–c_2_ highlight the POPD fiber like structures having a diameter between 112 and 650 nm and the length of in the range 4–42 µm. According to Figure 1a_2_–c_2,_ as the amount of PVDF in the mixture of the chemical polymerization reaction of OPD increases, a process of assembling PVDF spheres onto the surface of the POPD fiber like structures takes place.

**Figure 2:**
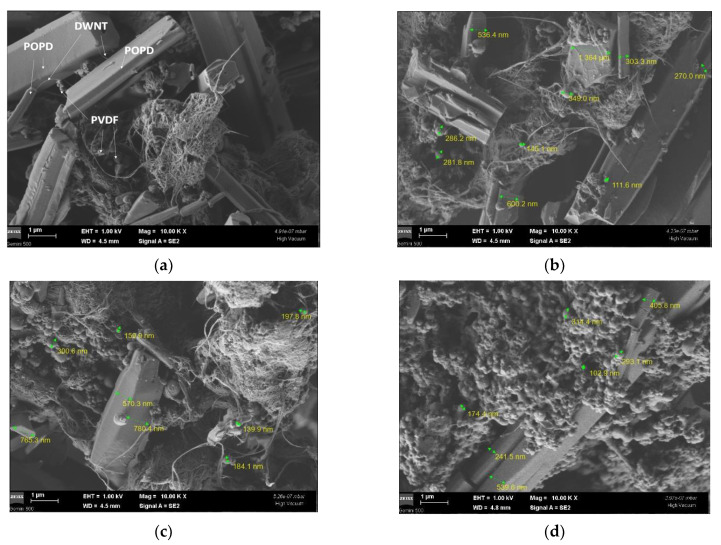
SEM images of the composites containing the POPD fiber like structures, PVDF spheres and DWNTs for which carbon nanotubes concentration is equal to 0.5 wt.% (the D sample, (**a**)), 1 wt.% (the E sample, (**b**)) and 2 wt.% (the F sample, (**c**)). (**d**) shows the SEM image of composite based on the POPD-PVDF blends and DWNTs, when the carbon nanotubes concentration is equal to 1 wt.% (the G sample, (**d**)).

**Figure 3:**
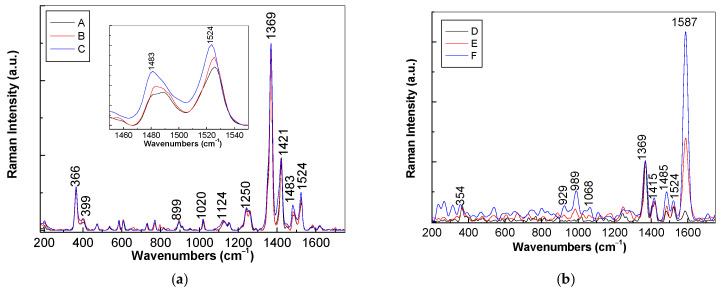
Raman spectra of the POPD-PVDF blends labeled as samples A (black curve, (**a**)), B (red curve, (**a**)) and C (blue curve, (**a**)) as well as the POPD-PVDF/DWNTs composites labeled as samples D (black curve, (**b**)), E (red curve, (**b**)) and F (blue curve, (**b**)).

**Figure 4:**
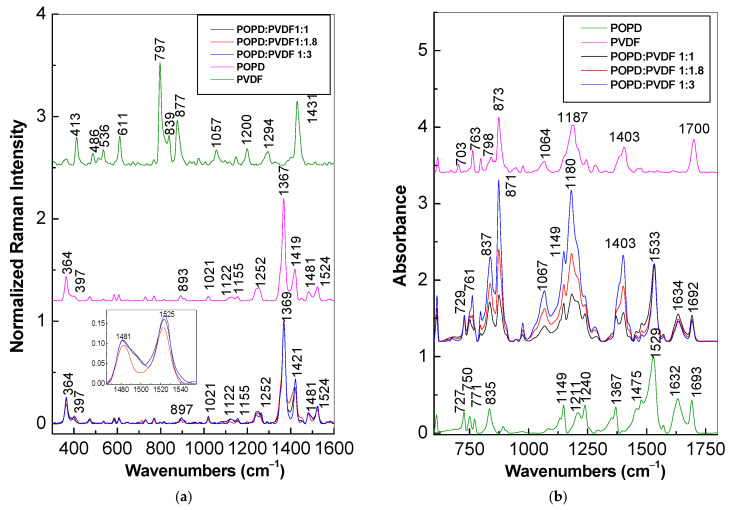
Raman (**a**) and IR (**b**) spectra of the samples labeled as A1, B1 and C1.

Figure 2 highlights the presence of DWNTs both onto the surface of the PVDF fiber like structures and the PVDF spheres. In order to support this statement in Figure 2a, we pointed with arrows to several POPD fiber like structures, PVDF spheres and DWNT bundles. In contrast with Figure 1b_1_,b_2_, where POPD fiber like structures with length ~5.5–42 µm and diameter of ~360–658 nm are observed, in Figure 2, the POPD fiber like structures have a diameter ~500–765 nm. In order to confirm that the morphological structures shown in Figure 1 and Figure 2 belong to POPD, PVDF and DWNTs, Figure 3 and Figure 4 show the Raman spectra and IR spectra of the A, B, C, D, E and F samples.

**Scheme 1:**
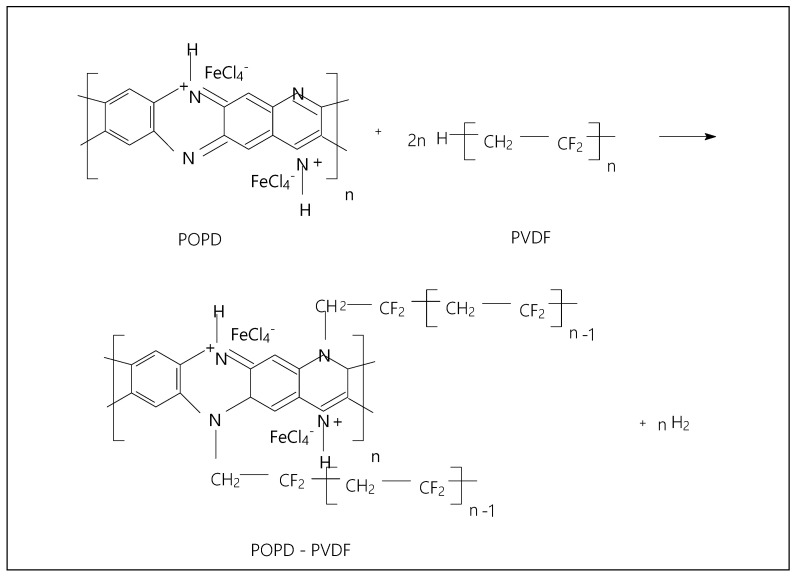
The chemical reaction of POPD doped with FeCl_4_^-^ ions with PVDF.

Regardless of the ratio between the concentrations of OPD and PVDF, the Raman spectra of the A, B and C samples (Figure 3a) are characterized by Raman lines peaked at 366, 889, 1250, 1369, 1421, and 1524 cm^−1^ which were assigned to the following vibrational modes of POPD: quinoid ring deformation, out of plane C-H bending in benzene nuclei of phenazine skeleton, C-N stretching in quinoid imide units, C-N^+^, phenazine like structure and C=N stretching in phenazine structure, respectively [31,32]. The Raman line peaked at around 399 cm^−1^ (Figure 3a) belongs to the T2 symmetrical vibrational mode of [FeCl_4_]^−^ ions, which compensates the positive charges existing on the macromolecular chain of POPD [33]. The Raman lines having maxima at 1020, 1124 and 1481–1483 cm^−1^ (Figure 3a) are not located far from the lines calculated by the density-functional theory which were reported to be situated at 1014, 1117 and 1477 cm^−1^; they were assigned to the following vibrational modes of α- PVDF: CH_2_ torsion (A_g_), symmetrical stretching of the C-C bond (A_u)_ and CH_2_ deformation + CH_2_ wagging (A_g_) [34,35]. According to Figure 3a, when increasing the PVDF weight in the mixture of the chemical polymerization reaction of OPD one observes: (i) an increase in the relative intensity of the Raman line situated in the spectral range 1430–1490 cm^−1^; (ii) a gradual shift of the Raman line from 1481 cm^−1^ (A sample) to 1483 cm^−1^ (B sample) and 1488 cm^−1^ (C sample); (iii) the ratio between the intensities of the Raman lines peaked at 1481–1488 and 1524 cm^−1^ varies from 0.55 (A sample) to 0.57 (B sample) and 0.67 (C sample). Taking into account that the Raman lines peaked at 1524 and 1481–1488 cm^−1^ are assigned to the vibrational modes of C=N stretching in phenazine structure of PVDF and CH_2_ deformation + CH_2_ wagging of PVDF, the gradual increase of the ratio between the intensities of these two lines (I_1524_/I_1481–1488_) form 0.55 (A sample) to 0.67 (C sample) indicates a higher weigh of the vibrational modes of PVDF in contrast with those of POPD. These results prove the increase of the PVDF weight in the A, B and C samples mass. In order to prove experimentally that the higher intensity of the vibrational modes of PVDF is not trivially due to the increase of the PVDF amount, Figure 4 shows the Raman and IR spectra of the samples prepared by the mixture of the two polymers. The mass of the two polymers is equal to: (i) 0.045 g POPD and 0.045 g PVDF; (ii) 0.05 g POPD and 0.09 g PVDF and (iii) 0.065 g POPD and 0.18 g PVDF, i.e., the POPD:PVDF weight ratio is 1:1 (A1 sample), 1:1.8 (B1 sample) and 1:3 (C1 sample). In contrast with Figure 3a, the analysis of Figure 4a indicates that: (i) the ratio between the intensities of the Raman lines peaked at 1481 and 1525 cm^−1^ shows small variations, i.e., this ratio is equal to 0.68, 0.67 and 0.64 in the case of the A1, B1 and C1 samples, respectively; and (ii) significant differences are observed in the case of the ratio between the intensities of the Raman lines peaked at 1369 and 1481 cm^−1^ (I_1369_/I_1481_) belonging to vibrational modes of POPD and PVDF; thus, the I_1369_/I_1481_ ratio has a value equal to 9.23 (Figure 4a) and 7.38 (Figure 3a) in the case of the C1 and C samples, i.e., when the two samples have the same weight ratio of the two polymers, they being prepared by the mixture methods of POPD and PVDF and the chemical polymerization of OPD in the presence of PVDF, respectively. These results confirm that the samples synthesized by the chemical polymerization of OPD in the presence of PVDF do not correspond to a mixture of POPD and PVDF, the product of the polymerization reaction being that shown in Scheme 1. Additional results, which confirm once again this statement, are shown in Figure 4b by IR spectroscopy.

**Figure 5:**
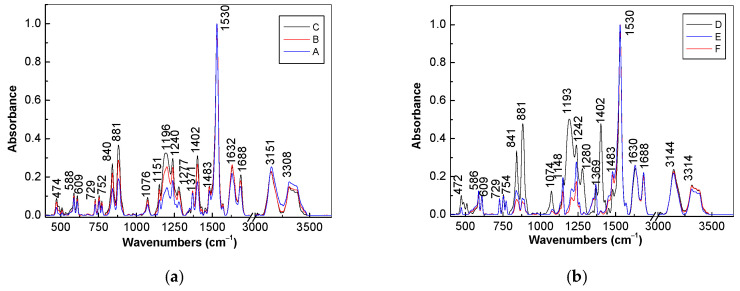
IR spectra of the samples labeled as samples A (black curve, (**a**)), B (red curve, (**a**)), C (blue curve, (**a**)), D (black curve, (**b**)), E (blue curve, (**b**)) and F (red curve, (**b**)).

In the case of the A1, B1 and C1 samples, namely when no interaction occurs during the mixing POPD with PVDF, the IR spectra should appear as a modulated sum of the contribution of the vibrational modes of the two polymers. According to Figure 4b, the IR spectrum of POPD shows an intense IR band with a maximum at 1529 cm^−1^ having a shoulder at 1475 cm^−1^, which is accompanied by other IR bands of low intensity [17], while the IR spectrum of PVDF shows four IR bands of high intensity having the maxima at 873, 1187, 1402 and 1703 cm^−1^ [36,37]. The IR spectra of the A1, B1 and C1 samples in Figure 4b correspond to the sum of the IR spectra of the two polymers. By increasing the PVDF weight in the A1, B1 and C1 samples, an increase in the absorbance of the IR bands of PVDF at 873, 1187, 1402 and 1703 cm^−1^ is seen. In comparison with the IR spectra of the A1, B1 and C1 samples (Figure 4b), in the cases of the IR spectra of the A, B and C samples are marked significant differences both in the absorbance of the IR bands of the PVDF and in the position of the IR bands localized in the spectral range 1000–1200 cm^−1^, when an up-shift of the IR bands from 1067 and 1180 cm^−1^ (Figure 4b) to 1076 and 1196 cm^−1^, respectively (Figure 5a) occurs. In our opinion, these differences are irrefutable proofs that the A, B and C samples, prepared by the chemical polymerization of OPD in the presence of PVDF, cannot be assimilated as being similar to the samples obtained by mixing the two polymers. The chemical polymerization of OPD in the presence of PVDF involves a chemical reaction, which results in compounds like that shown in Scheme 1.

Returning to Figure 3, regardless of the PVDF weight in the mixture of the chemical polymerization reaction of OPD, the ratio between the intensities of the Raman lines peaked at 1369 and 1421 cm^−1^ is equal to ~2.6. This shows that there is no change in the doped state of POPD. The presence of DWNTs in the mixture of the chemical polymerization reaction of OPD in the presence of PVDF induces a down-shift of the Raman line from 1421 cm^−1^ (Figure 3a) to 1415 cm^−1^ (Figure 3b). This result indicates a covalent functionalization of DWNTs with the POPD-PVDF blends via a phenazine like structure. When increasing the DWNTs weight in the mixture of the chemical polymerization reaction of OPD in the presence of PVDF, one observes in Figure 3b the following changes: (i) an increase in the relative intensity of the Raman line peaked at 1587 cm^−1^, assigned to the tangential mode (TM) of the DWNTs so that the ratio between the intensities of the Raman lines from 1369 and 1587 cm^−1^ varies from 5.57 (D sample) to 0.72 (E sample) and 0.32 (F sample); (ii) a decrease of the ratio between the intensities of the Raman lines peaked at 1369 and 1415 cm^−1^ from 3.03 (D sample) to 2.94 (E sample) and 2.51 (F sample); (iii) an increase of the ratio between the intensities of the Raman lines peaked at 1483–1485 and 1521 cm^−1^ from 0.75 (D sample) to 1 (E sample) and 1.42 (F sample). In our opinion: (i) the increase of the intensity of the Raman line assigned to TM of the DWNTs confirms the presence of an increasing amount of carbon nanotubes in the case of the D, E and F samples; (ii) the decreasing the ratio between the intensities of the Raman lines at 1369 and 1415 cm^−1^, assigned to the vibrational modes of the C-N^+^ bond and phenazine like structure [31,32] indicates a partial de-doping of POPD; (iii) the increasing ratio between the intensities of the Raman lines at 1483–1485 and 1521 cm^−1^, assigned to the vibrational modes of CH_2_ deformation + CH_2_ wagging (A_g_) of PVDF [34,35] and C=N stretching in phenazine structure [31,32] indicates that a chemical interaction between POPD and DWNTs takes place. A consequence of this interaction is the decrease of the weight of the C=N bond in the favor of C-N. These results can be understood if we accept that a reaction between POPD doped with FeCl_4_^−^ ions with DWNTs takes place.

Additional information concerning vibrational properties of the A, B, C, D, E and F samples are reported by IR spectroscopy. According to Figure 5, one observes that all IR spectra are characterized by IR bands with maxima at: (i) 609, 840, 881, 1076–1074, 1196–1193, 1402 cm^−1^, which belong to PVDF, these being attributed to the vibrational modes of the deformation of CF_2_ + CCC in α-PVDF, the rotation of CH_2_ bond + anti-symmetrical stretching CF_2_ in β-PVDF, anti-symmetrical stretching CC + CF_2_ in β-PVDF, anti-symmetrical stretching CC in β-PVDF + wagging CF_2_ + CH_2_, anti-symmetrical stretching CF_2_ + wagging CH_2_ in α-PVDF and wagging CH_2_ + anti-symmetrical CC in β-PVDF [36,37]; and (ii) 472–588, 729–754, 1151–1148, 1240–1242, 1371–1369, 1277–1280, 1531–1529, 1632–1630 and 1688 cm^−1^, which belong to POPD, these being assigned to the vibrational modes of aromatic ring deformation, C-H out-of-plane bending in benzene rings of the phenazine skeleton, in-plane C-C-H bending in benzene rings, C-N=C stretching in the benzenoid units, C-N stretching in quinoid imide units, C-NH-C, C=NH^+^-C, C=C stretching in benzene ring + C-C stretching in quinoid ring and ammonium end-groups in macromolecular chain [37,38].

The IR bands at 3151 and 3308 cm^−1^ belong to the vibrational mode of stretching of NH with intramolecular association and intramolecular bond, respectively, of POPD [38]. A careful analysis of Figure 5a indicates that as increasing the PVDF weight in the mass of the POPD-PVDF blends (labeled as the A, B and C samples) takes place: (i) a gradual increasing in absorbance of the IR bands localized at 840, 881, 1196 and 1402 cm^−1^ belonging to PVDF; (ii) the ratio between the absorbance of IR bands at 881 and 840 cm^−1^ is equal to 1.37 (A sample), 1.27 (B sample) and 1.36 (C sample); and (iii) the ratio between the absorbance of the IR band of PVDF situated at 1196 cm^−1^ and that of the IR bands of POPD localized at 1240 and 1277 cm^−1^ (A_1196_:A_1240_:A_1277_) varies from 1:1.26:0.49 (A sample) to 1:0.99:0.5 (B sample) and 1:0.91:0.46 (C sample). The decrease in the absorbance of the IR band at 1240 cm^−1^ indicates a diminution of the weight of the C-N=C vibrational modes of POPD. This fact can be explained only if PVDF is covalently bonded on the POPD macromolecular chain. A puzzling fact is that an increase in the absorbance of the IR bands at 840 and 1402 cm^−1^ was also reported in the case of the composites of the type PVDF/single-walled carbon nanotubes when a covalent functionalization of carbon nanotubes with PVDF was reported [38]. Another fact which indicates a chemical interaction between POPD and PVDF regards the value of the ratio between the absorbance of IR bands peaked at 881 and 840 cm^−1^ whose value is lower in the case of A, B and C samples, i.e., 1.37, 1.27 and 1.36, than that reported in the case of PVDF (3.35) [39]. Summarizing the above variations and taking into account (i) the decrease in the absorbance of the IR band at 1240 cm^−1^, (ii) the increase in the absorbance of the IR bands at 840 and 1402 cm^−1^ and (iii) the smaller values of the ratio between the absorbance of IR bands peaked at 881 and 840 cm^−1^, we conclude that during the chemical polymerization of OPD in the presence of PVDF, the generation of new covalent bonds of the type N-CH between the two macromolecular compounds takes place according to Scheme 1.

**Scheme 2:**
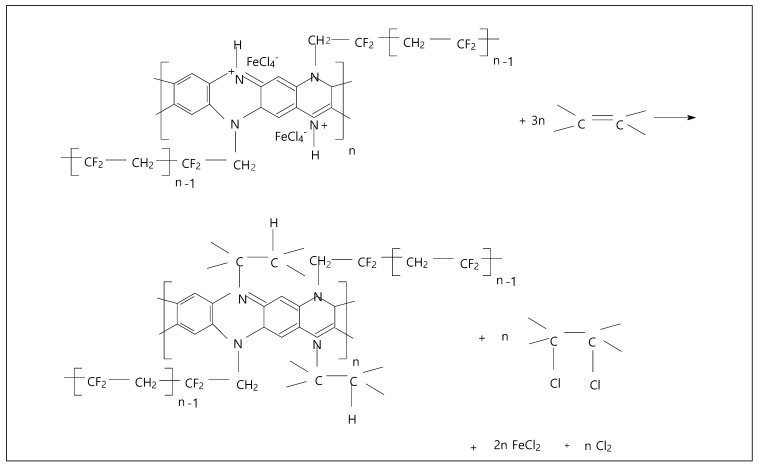
The chemical reaction of POPD doped with FeCl_4_^–^ ions with DWNTs.

**Scheme 3:**
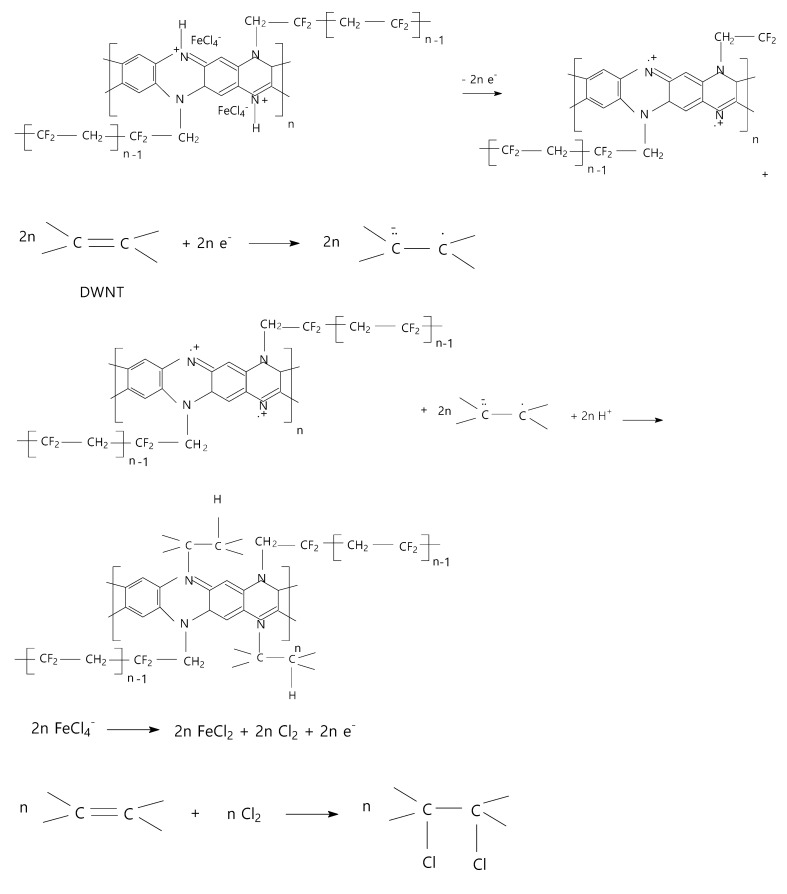
The chemical mechanism of the interaction of POPD doped with FeCl_4_^-^ ions with DWNTs.

In comparison with the IR spectrum of the B sample, the following changes are reported in the IR spectra of the D, E and F samples: (i) a change of the ratio between the absorbance of the IR bands having the peaks at 1193, 1242 and 1280 cm^−1^; this ratio is equal to: (i) 1:0.72:0.48 in the case of the D sample; (ii) 1:2.08:0.22 in the case of the E sample and (iii) 1:2.12:0.5 in the case of the F sample; (ii) a down-shift of the IR band from 3151 cm^−1^ (Figure 5a) to 3144 cm^−1^ (Figure 5b); (iii) an up-shift of the IR band from 3308 cm^−1^ (Figure 5a) to 3314 cm^−1^ (Figure 5b); and (iv) a variation in the ratio between the absorbance of the IR bands localized at 1242 and 1530 cm^−1^, from 0.36 (D sample) to 0.27 (E sample) and 0.17 (F sample). This decrease of the ratio between the absorbance of the IR bands having the peaks at 1242 and 1530 cm^−1^, indicates that the interaction of POPD in doped state with DWNTs leads to a composite in which the share of C-N-C bonds is higher than that existing in the doped state of POPD. This result is in good agreement with the reaction of the blend PVDF-POPD in a doped state with DWNT, which is shown in Scheme 2. The reaction, shown in Scheme 2, takes into account the property of carbon nanotubes to accept electrons when resulting in anion radical species that are unstable and react with compounds in the reaction medium. The chemical mechanism of the reaction shown in Scheme 2 is detailed in Scheme 3.

The second product of Scheme 2 corresponds to DWNTs on the surface of which part from the C = C bonds has been transformed into C-Cl bonds. Evidence for the new bonds of the types C-Cl and C-H is highlighted in Figure 3b, where Raman lines peaked at 829, 989 and 1068 cm^−1^ are observed, which were also reported in the case of dichlorobenzene [40]. The Raman lines at 829, 989 and 1068 cm^−1^ are assigned to the vibrational modes A_2_ out-of-plane of the aromatic ring, A_1_ in-plane of the C-H stretching + aromatic ring and A_1_ in-plane of the C-Cl bond, respectively [36].

**Figure 6:**
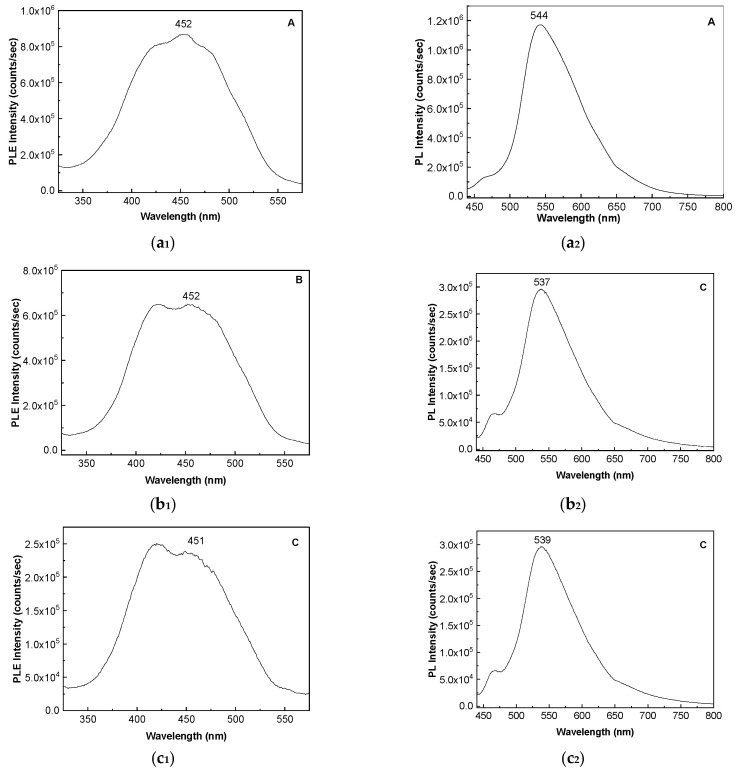
PLE (1) and PL (2) spectra of the POPD-PVDF blends labeled as samples A (**a_1_**,**a_2_**), B (**b_1_**,**b_2_**) and C (**c_1_**,**c_2_**), recorded at the emission wavelength of 600 nm and the excitation wavelength of 420 nm, respectively.

Figure 6 highlights that in the case of the POPD-PVDF blends, as increasing the PVDF weight in the mass of the A, B and C samples takes place: (i) a gradual decrease in the intensity of the PLE band with the maximum at 452 nm from 8.66 × 10^5^ counts/sec (A sample) to 6.47 × 10^5^ counts/sec (B sample) and 2.38 × 10^5^ counts/sec (C sample); (ii) a significant decrease in the intensity of the PL band localized in the spectral range 450–750 nm from 1.17 × 10^6^ (A sample) to 4.76 × 10^5^ counts/sec (B sample) and 2.96 × 10^5^ counts/sec (C sample). In the context of these variations, we note that: (i) PVDF does not show PL, when the excitation wavelength is equal to 420 nm; and (ii) a PL band localized in the spectral range 450–750 nm was recently reported in the case of POPD [41]. The above variations indicate that a POPD PL quenching process is induced in the presence of PVDF.

**Figure 7:**
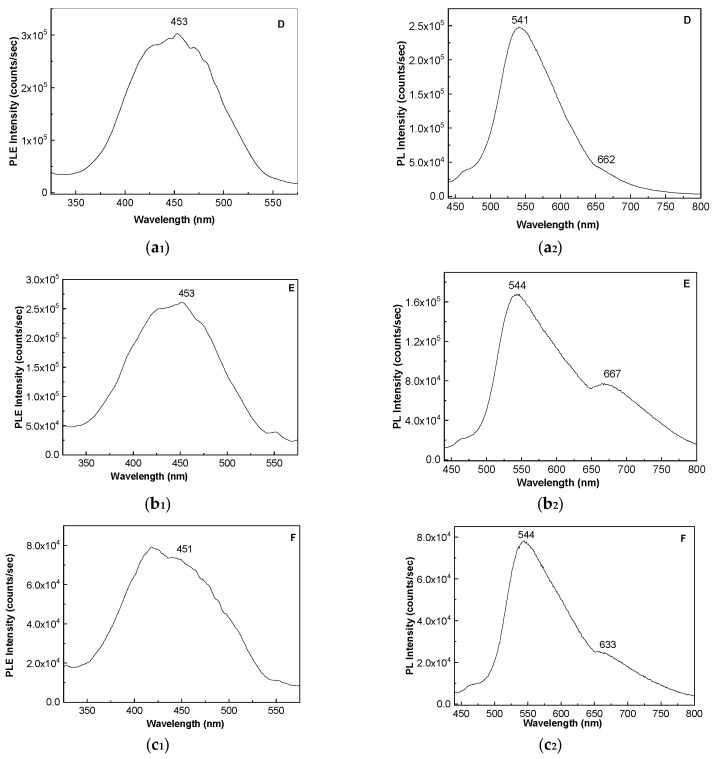
PLE (1) and PL (2) spectra of the composites based on the POPD-PVDF blends and DWNTs labeled D (**a_1_**,**a_2_**), E (**b_1_**,**b_2_**) and F (**c_1_**,**c_2_**) recorded at the emission wavelength of 600 nm and the excitation wavelength of 420 nm, respectively.

According to Figure 7, additional changes both in the intensity and in the shape of the PL spectra are induced to the POPD-PVDF blends in the presence of DWNTs. In contrast to sample B, the following variations of the PL and PLE spectra are observed to be induced as a consequence of the addition of an increasing amount of DWNTs in the mass of the polymerization reaction of OPD and PVDF: (i) a decrease in the intensity of the PLE band situated in the spectral range 350–550 nm from 6.47 × 10^5^ counts/sec (B sample, Figure 6b_1_) to 3.03 × 10^5^ counts/sec (D sample, Figure 7a_1_), 2.6 × 10^5^ counts/sec (E sample, Figure 7b_1_) and 7.17 × 10^4^ counts/sec (F sample, Figure 7c_1_); (ii) a progressively decrease in the intensity of the PL band with the peak at 541–544 nm from 4.76 × 10^5^ counts/sec (B sample, Figure 6b_2_) to 2.48 × 10^5^ counts/sec (D sample, Figure 7a_2_), 1.67 × 10^5^ counts/sec (E sample, Figure 7b_2_) and 7.8 × 10^4^ counts/sec (F sample, Figure 7c_1_); and (iii) the increase in the intensity of the emission band situated in the spectral range 650–800 nm, whose intensity increases as the DWNTs amount varies from 5 mg to 10 mg and 20 mg in the mixture of the chemical polymerization reaction of the D, E and F composites. These results clearly demonstrate the role of DWNTs as POPD PL quenching agents.

**Figure 8:**
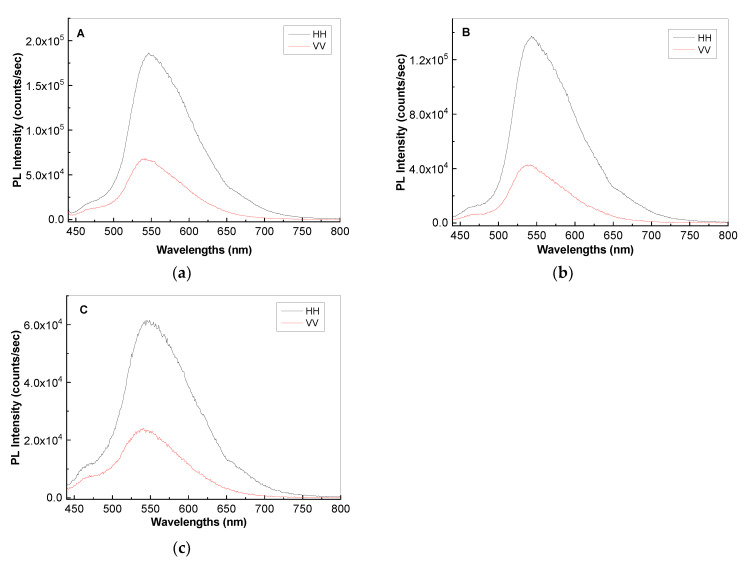
Polarized PL spectra of the POPD-PVDF blends labeled as A (**a**), B (**b**) and C (**c**) samples. All PL spectra were recorded at the excitation wavelength equal to 420 nm.

**Figure 9:**
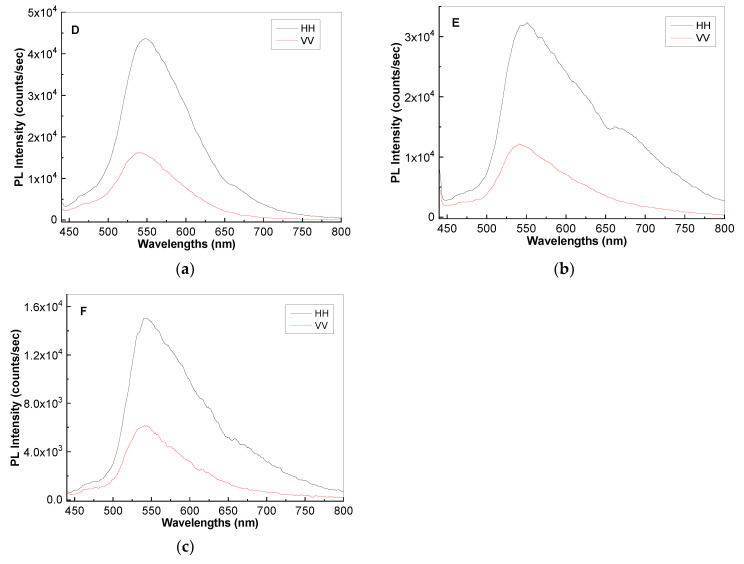
Polarized PL spectra of the composite materials based on the POPD-PVDF blends and DWNTs labeled with D (**a**), E (**b**) and F (**c**). All PL spectra were recorded at the excitation wavelength equal to 420 nm.

In order to better understand the orientation of the macromolecular chains of POPD and PVDF in the absence and presence of DWNTs, Figure 8 and Figure 9 show the anisotropic PL spectra of the A, B, C, D, E and F samples. Thus, Figure 8 and Figure 9 highlight the vertical (V) and horizontal (H) polarization PL spectra when the V and H excitation beams were used. Depending on how the polarizers are mounted at excitation and emission in the spectrophotometer and the calibration factor of the instrument (*G)*, the values of the anisotropy (*r*) and angle of binding of the two macromolecular compounds (θ) [42], can be calculated with the following equations:

$$r=\;\frac{I_{VV}-GI_{VH}}{I_{VV}+2GI_{VH}}$$

$$r=0.4⟦\left(3\;cos^{2}\theta -1\right)/2⟧$$

$$G=\frac{I_{HV}}{I_{HH}}$$

where: *I_VV_* and *I_HH_* correspond to the intensity of the light when the excitation and emission polarizers were mounted vertically and horizontally, respectively, in spectrophotometer; *I_VH_* and *I_HV_* correspond to the intensity of the light when the excitation/emission polarizers were mounted vertically/horizontally and horizontally/vertically, respectively, in the spectrophotometer. Using above equations, the following values of r and θ were calculated in the case of the samples labeled: (i) A, 0.070 and 47.9°; (ii) B, 0.046 and 50.2°; (iii) C, 0.041 and 50.7°; (iv) D, 0.094 and 45.6°; (v) E, 0.172 and 38.1° and vi) F, 0.144 and 40.8°.

These results in the case of the A, B and C samples highlight that regardless of the weight of the PVDF spheres onto the POPD fiber like structures surface, a variation in θ of only 2.8° occurs. In comparison with the B sample, the presence of DWNTs in the D, E and F composites induces a decrease in θ of ~12°. This behavior can be explained by taking into account the π-π stacking bonds established between the semi-oxidized structure of POPD and the graphitic structures of DWNTs when variations in the excitation and emission transition dipoles take place.

Summarizing the above results, the main improvements reported in this work concerning the morphological and photoluminescent properties of the POPD fiber like structures in the case of the POPD-PVDF blends and their composites with DWNT versus those of the type POPD-PEO and POPD-PEO/DWNTs [41] consist of: (i) increasing the length of POPD fiber like structures, from ~1–20 μm [41] to 4–42 μm, simultaneous with decreasing their diameter from 256–790 nm to 112–650 nm, when at the chemical polymerization of OPD instead of PEO [41] is added PVDF; (ii) the intensity of the photoluminescence of the POPD fiber like structures is higher, when these are obtained by chemical polymerization of OPD in the presence of PVDF (4.76 × 10^5^ counts/sec in the case of the B sample) in comparison with PEO (1.3 × 10^4^ counts/sec) [41]; and (iii) the intensity of the photoluminescence of the POPD-PVDF/DWNTs composites (3.03 × 10^5^ counts/sec in the case of the D sample) is higher in comparison with those reported in the case of the POPD-PEO/DWNTs composites (~5.6 × 10^4^ counts/sec) [41].

## 3. Materials and Methods

PVDF, o-phenylenediamine (OPD), FeCl_3_, N, N’-dimethylformamide (DMF) and DWNTs were purchased from Sigma-Aldrich (St. Louis, MO, USA).

In order to obtain the blends based on PVDF spheres and POPD fiber like structures, the following protocol was used. In the first step, an aqueous solution of OPD (0.09 g in 20 mL H_2_O) and three anhydrous solutions of PVDF in DMF having concentrations equal to 0.045 g/mL, 0.09 g/mL and 0.225 g/mL were prepared. After mixing the solution of OPD in H_2_O with the three solutions of PVDF in DMF, a solution of FeCl_3_ (2.34 mL, 0.71 M) was added to each mixture, after which it was stirred at room temperature for 5 min. resulting in a solution with a brown–red color. This reaction mixture was stored for 2 h and after that, this was filtered, washed with distilled water and then dried at 50 °C under vacuum for 24 h. As shown below, using the ratio between the concentrations of OPD and PVDF equal to 0.1, 0.05 and 0.02, the POPD-PVDF blends containing the fiber like structures of POPD doped with FeCl_4_^−^ ions and the PVDF spheres, labeled in the following as the A, B and C samples resulted. The weight of the POPD-PVDF blends labeled as the A, B and C samples, resulted according to the above protocol, is equal to ca. 0.09 g, 0.14 g and 0.24 g. Taking into account the mass of PVDF added to the chemical polymerization reaction of OPD, we estimate that the mass of the two polymers in the case of: (i) the A sample is 0.045 g POPD and 0.045 g PVDF; (ii) the B sample is 0.05 g POPD and 0.09 g PVDF and (iii) the C sample is 0.06 g POPD and 0.18 g PVDF. The difference between the samples resulted from the chemical polymerization of the OPD monomer in the presence of PVDF and the mixtures of the two polymers, POPD and PVDF, prepared using the weight estimated to be in the A, B and C samples was highlighted by Raman scattering and IR spectroscopy.

In order to synthesize composites based on POPD, PVDF and DWNTs, we have used the above protocol, the only difference being the adding of various DWNTs weights, i.e., 5, 10 and 20 mg, in the mixture of the OPD/PVDF solutions, having the ratio between the concentrations of OPD and PVDF equal to 0.05. The resulted composites were labeled as the D, E and F samples. A composite labeled as the G sample, having the ratio between the concentrations of OPD and PVDF equal to 0.02 to which was added 10 mg of DWNTs, was also prepared.

In order to highlight the morphology of the A, B and C blends, as well as the D, E, F and G composites, SEM images were recorded with a Zeiss Gemini 500 scanning electron microscope (Zeiss, Oberkochen, Germany). SEM analyses were performed on samples as prepared in high vacuum mode with the secondary electron detector (SE2) and an acceleration voltage of 1kV.

The vibrational properties of all samples prepared in this work were recorded with (i) an FT Raman spectrophotometer, Multiram model, endowed with a YAG:Nd laser and (ii) an FTIR spectrophotometer, Vertex 80 model, both purchased from Bruker (Billerica, MA, USA).

The photoluminescence (PL) and photoluminescence excitation (PLE) spectra of all samples, prepared in this work, were recorded with a Fluorolog-3 spectrophotometer, FL-3.2.1.1 model, endowed with a Xe lamp, purchased from Horiba Jobin Yvon (Palaiseau, France).

## 4. Conclusions

In this work, we have reported new results concerning the optical properties of the composites based on POPD fiber like structures, PVDF spheres and DWNTs. Using scanning electron microscopy (SEM), Raman scattering, IR spectroscopy and photoluminescence we have demonstrated that: (i) the chemical polymerization of o-phenylenediamine in the presence of PVDF leads to the blends containing the PVDF spheres and the POPD fiber like structures; (ii) the chemical polymerization reaction of o-phenylenediamine in the presence of PVDF induces a decrease in the absorbance of the IR band at 1240 cm^−1^ assigned to the C-N = C vibration mode of POPD, as a consequence of generation of new C-N bonds between the two macromolecular compounds; (iii) the addition of DWNTs in the mixture of the chemical polymerization reaction of o-phenylenediamine in the presence of PVDF leads to composites of the type DWNTs covalent functionalized with the POPD-PVDF blends, a chemical mechanism being proposed in this paper (Scheme 3); (iv) the PVDF spheres induce a photoluminescence (PL) quenching process of the POPD fiber like structures; in the presence of DWNTs, a PL quenching process of the POPD-PVDF blends is reported; and (v) according to the anisotropic photoluminescence studies, a variation of the angle of binding of the two macromolecular compounds with ca. 12° is reported to be induced by DWNTs.

## Data Availability

Samples are available from authors.
